# Prevalence of COVID-19 in 10,000 samples from 7853 patients in Eastern Turkey by positive real-time PCR

**DOI:** 10.2217/fmb-2020-0134

**Published:** 2021-07-05

**Authors:** Murat Karamese, Didem Ozgur, Ceyda Tarhan, Susamber D Altintas, Okan Caliskan, Aysegul Tuna, Saliha Kazci, Mursel Karadavut, Abdullah Gumus, Gozde Apaydin, Necati Mumcu, Onur Coruh, Emin E Tutuncu

**Affiliations:** 1 Department of Medical Microbiology, Kafkas University, Faculty of Medicine, Kars, 36100, Turkey; 2 Department of Otorhinolaryngology, Iğdır State Hospital, Iğdır, 76000, Turkey; 3 Department of Chest Diseases, Iğdır State Hospital, Iğdır, 76000, Turkey; 4 Department of Infectious Disease & Clinical Microbiology, Harakani State Hospital, Kars, 36100, Turkey; 5 Department of Infectious Disease & Clinical Microbiology, Ardahan State Hospital, Ardahan, 75100, Turkey; 6 Department of Internal Medicine, Ardahan State Hospital, Ardahan, 75100, Turkey; 7 Department of Pediatric Health & Diseases, Iğdır State Hospital, Iğdır, 76000, Turkey; 8 Department of Infectious Disease & Clinical Microbiology, Iğdır State Hospital, Iğdır, 76000, Turkey; 9 Department of Anesthesiology & Reanimation, Ardahan State Hospital, Ardahan, 75100, Turkey; 10 Department of Infectious Disease & Clinical Microbiology, Kafkas University, Faculty of Medicine, Kars, 36100, Turkey

**Keywords:** COVID-19, positivity rate, prevalence, real-time PCR, SARS-CoV-2

## Abstract

**Aim:**

COVID-19, caused by SARS-CoV-2, started in December 2019 and has spread across the world.

**Materials & methods:**

We analyzed real-time PCR results of 10,000 samples from 2 April to 30 May 2020 in three neighbor cities located in the East of Turkey. The final study population was 7853 cases, after excluding screening tests.

**Results:**

Real-time PCR was performed to detect the SARS-CoV-2-specific RNA-dependent-RNA-polymerase gene fragment. The number of total positive samples out of 7853 were 487; however, the number of nonrepeating positive patient was 373 (4.8%). Cough and fever were the most common symptoms in positive cases.

**Conclusion:**

Epidemiologic studies should be performed about the prevalence of SARS-CoV-2 infection to better understand the effect of the virus across the world.

COVID-19, which is caused by SARS-CoV-2, started in December 2019 and has spread across the world [1,2]. The disease is characterized by high fever, cough, shortness of breath, pneumonia, loss of the sense of smell/taste and other respiratory tract symptoms and became a great global public health concern [3]. The real-time PCR (RT-PCR) analysis was used to detect the SARS-CoV-2 virus from respiratory specimens such as tracheal aspirate, nasopharyngeal swab and sputum [4]. On the other hand, some physicians argued that computed tomography (CT) imaging may help the identification of SARS-CoV-2 infection, even if the RT-PCR results were negative [5,6]. From now on, RT-PCR is a gold-standard diagnostic method and has provided more accuracy and quick diagnosis for SARS-CoV-2 detection within nearly 2 h [4].

In this study, we retrospectively analyzed RT-PCR results of 10,000 samples from 2 April to 30 May 2020 in Kars, Iğdır and Ardahan, cities that are located in the East of Turkey. After the screening tests were excluded, the final study population was 7853 cases. All the cases were suspected of SARS-CoV-2 infection because of symptoms or close contact with a COVID-19 patient. RT-PCR was performed to detect the SARS-CoV-2-specific RNA-dependent RNA polymerase gene fragment from respiratory specimens such as tracheal aspirate, nasopharyngeal swab and sputum. The main purpose of this study was to share the prevalence data of SARS-CoV-2 positivity and add to the current literature.

## Materials & methods

### Data collection

**Figure 1.**
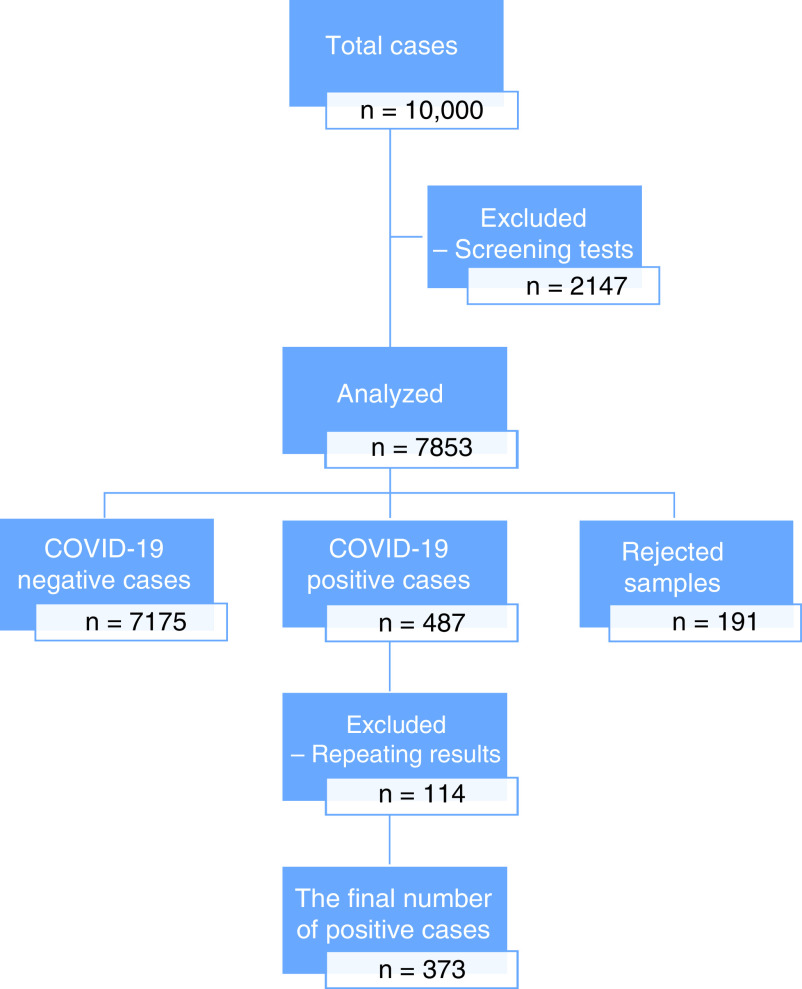
Study flow diagram.

The study was approved by both the Republic of Turkey Ministry of Health COVID-19 Scientific Research Evaluation Commission (approval date: 2 May 2020; number: 2020-05-03T18_02_30) and the Local Ethics Committee of Kafkas University Faculty of Medicine (approval date: 6 May 2020 number: 80576354-050-99/129). A total of 10,000 samples from 2 April to 30 May were tested for SARS-CoV-2 infection in Kars, Iğdır and Ardahan, cities that are located in the East of Turkey; however, 7853 cases were evaluated who had typical respiratory infection symptoms such as fever, cough and shortness of breath or close contact with a COVID-19 patient. The patients who had any symptoms of COVID-19 disease had a RT-PCR SARS-CoV-2 test. The samples were collected day by day between 1 April and 29 May and unsuitable, flowed and leaked samples were rejected. In this study, the screening RT-PCR test results (n = 2147) were excluded (Figure 1). All RT-PCR tests from three cities were performed at Kafkas University, Faculty of Medicine, Department of Medical Microbiology, Molecular Microbiology Laboratory, Kars, Turkey.

### RT-PCR protocol

The detection of SARS-CoV-2 virus in respiratory specimens especially in nasopharyngeal swabs was detected by RT-PCR technique via using Roche Lightcycler-96 device (Roche Diagnostic Systems, Basel, Switzerland). RT-PCR was performed using SARS-CoV-2 (2019-nCoV) qPCR Detection Kit (Bioeksen R&D, İstanbul, Turkey) that is targeting SARS-CoV-2-specific RNA-dependent RNA polymerase gene fragment. The final PCR concentration was 20 μl (10 μl qPCR master mix, 5 μl primer/probe set and 5 μl template). The nucleic acid amplification was performed with the following PCR steps: reverse transcription stage (45°C, 15 min, one cycle), initial activation stage (95°C, 3 min, one cycle) and amplification stage (denaturation: 95°C, 5 s, annealing and extension: 55°C, 35 s, 45 cycles). All samples were run together with a SARS-Cov-2-positive control and -negative control (H_2_O). For data analysis, 2-^ΔΔ^ CT method was used and CT values were less than 40 was defined as a positive test.

### Statistical methods

Statistical analysis was performed with the statistical package for the Social Sciences version 22.0 (SPSS, IBM, NY, USA). The ‘number,’ ‘percentage,’ 'mean’ and ‘standard deviation’ were used for the descriptive statistics of the continuous variables. The independent samples *t*-test or Mann–Whitney *U*-test were used to compare two independent groups. The Pearson Chi-Square or Fisher’s exact tests were used to analyzing categorical data. The results were evaluated with a CI of 95% and the level of significance, p, was set at 0.05.

## Results

A total of 7853 cases were included from three neighboring cities. Of those, 4642 (59.1%) were male and 3211 (40.8%) were female. The mean age of all cases was 38.14 ± 21.10. The nasopharyngeal swab samples (n = 7826, 99.7%) were the most preferred respiratory specimens. Detailed descriptive characteristics of all patients are in Table 1. The number of total positive samples was 487; however, the number of nonrepeating positive patient was 373 (4.8%). The distribution of nonrepeating positive patients was as follows: Kars (n = 181, 48.5%), Iğdır (n = 147, 39.4%) and Ardahan (n = 45, 12.1%). Of those, 156 (41.8%) were male and 217 (58.2%) were female. The mean age of positive cases was 36.96 ± 20.25. Cough and fever were the most common symptoms in positive cases (n = 111, 29.8% and n = 98, 26.3%, respectively); however, most of the positive cases were asymptomatic (n = 198). Additionally, respiratory diseases such as asthma and chronic obstructive pulmonary disease were the most recorded additional diseases for positive cases. Detailed information about the positive cases are in Table 2. The statistical analysis showed that there was a significant difference between positive and negative cases regarding gender (X^2^ = 50.903; p = 0.000), while no statistically significant difference was detected between them where age was concerned (Z = -0.980; p = 0.327).

**Table 1.** The descriptive characteristics of all patients from three neighboring cities.

Parameters	Kars		Iğdır		Ardahan		Total
	n	%	n	%	n	%	n
Samples (n)	3509	44.7	3130	39.9	1214	15.4	7853
**Gender**							
Male	2120	60.9	1758	56.2	764	62.9	4642
Female	1389	39.1	1372	43.8	450	37.1	3211
Mean age	38.54 ± 21.48		35.23 ± 19.80		44.33 ± 21.77		38.14 ± 21.10
Median age	34		31		41		33
**Age range**							
0–20	596		575		120		1291
21–40	1516		1539		494		3549
41–60	753		637		302		1692
60+	644		379		298		1321
**Specimen types**							
Nasopharyngeal swab	3506	99.9	3130	100	1190	98.0	7826
Sputum	0	0	0	0	24	2.0	24
Tracheal aspirate	3	0.1	0	0	0	0	3
**Results**							
Total positive	210	5.98	228	7.3	49	4.0	487
Negative	3243	92.4	2797	89.3	1135	93.8	7175
Rejected	56	1.6	105	3.5	30	2.5	191
Nonrepeating positives	181	5.15	147	4.7	45	3.70	373

**Table 2.** The descriptive characteristics of positive patients from three neighbor cities.

Characteristics	Positive cases (n = 373)	
	n	%
**Distribution of cases**		
Kars	181	48.5
Iğdır	147	39.4
Ardahan	45	12.1
**Gender**		
Male	156	41.8
Female	217	58.2
Mean age	36.96 ± 20.25	
Median age	33	
**Age range**		
0–20	70	18.8
21–40	152	40.8
41–60	108	28.9
60+	43	11.5
**Specimen types**		
Nasopharyngeal swab	370	99.2
Sputum	0	0
Tracheal aspirate	3	0.8
**Symptoms**		
Cough	111	29.8
Fever	98	26.3
Sore throat	49	13.2
Shortness of breath	43	11.5
	40	10.7
Headache	38	10.2
Myalgia	18	4.8
Stomachache	7	1.9
Diarrhea	6	1.6
Vomiting	6	1.6
Asymptomatic	198	53.1
**Additional diseases**		
Respiratory diseases	29	7.8
Hypertension	17	4.6
Cardiovascular diseases	12	3.2
Diabetes	9	2.4
Cancer	4	1.1

## Discussion

To our knowledge, this is one of the first epidemiologic studies about the RT-PCR positivity of suspected SARS-CoV-2 cases in our country. In 60 days, we studied 10,000 specimens to detect SARS-CoV-2; however, the screening tests were excluded (n = 2147). The positivity rates may vary depending on the study population and location. A study reported that 38% of 4880 cases were detected positive by RT-PCR [4]. Similarly, another study reported that the positivity rate was 51% [7]. The positivity rate of our population was 4.75%, which is almost compatible with the national positivity rates announced every day by the Minister of Health [8].

Pan *et al.* performed an epidemiologic study with 32,583 laboratory confirmed COVID-19 cases and reported that 51.6% of cases were female [9]. The percentages of positive male and female are parallel with the current literature. Among the 3211 female and 4642 male cases, females had a higher rate (n = 217, 6.75%) of confirmed cases compared with males (n = 156, 3.36%). This is parallel with the current literature. Although, the number of tested males was more than one and a half times the number of tested females, the positivity rate in females was nearly twofold that of males (6.75 vs 3.36%). In our study, most positive cases were in the 21–40 (40.8%) and 41–60 (28.9%) age ranges, respectively. Similarly, a study from China performed with 44,672 cases indicated that most positive cases were in the 30–79 (87%) age range [10]. The average range was generally above 55 years [7]; however, in our study data the average age was roughly 38 years. This may be due to the inclusion of a large number of asymptomatic and close-contact patients who were younger.

On the other hand, cough and fever were the most seen symptoms in positive cases, respectively; however, most of the cases were those who had close contact with a COVID-19 patient (asymptomatic patients). Most of the studies reported that the number of asymptomatic patients were high, and the most common symptoms were fever and cough [3,11,12]. Interestingly, total positive percentage was associated with gender but not age, in our study. By contrast, a study reported that SARS-CoV-2 infection was statistically related with both age and gender [4].

## Conclusion

In conclusion, epidemiologic studies should be performed about the prevalence of SARS-CoV-2 infection to better understand the effect of the virus around the world. On the other hand, prevalence of PCR positives among close contact screening and age and sex distribution may reflect healthcare access or behavior and this should be analyzed in future studies.

## Study limitations

Our study has some limitations. First, this was a retrospective study. Second, more detailed information should have been obtained because there were relatively large amounts of missing data for some outcomes. Third, the pandemic is still ongoing and the data about SARS-CoV-2 positivity could change; however, we wanted to share the suspected patients’ rates before the screening test had been started in our country. Fourth, information such as outpatient/inpatient was not available. Finally, the asymptomatic patients were not followed up in our study. The main aim was to present the prevalence of RT-PCR positivity of suspected COVID-19 patients. Further studies about the CT value difference of symptomatic and asymptomatic patients with a large population should be performed.

**Summary points**

- We analyzed real-time PCR results of 10,000 samples from 2 April to 30 May 2020 in three neighboring cities located in the east of Turkey.
- Real-time PCR was performed to detect the SARS-CoV-2-specific RNA-dependent-RNA-polymerase gene fragment.
- Cough and fever were the most common symptoms in positive cases.
- Epidemiologic studies should be performed about the prevalence of SARS-CoV-2 infection to better understand the effect of the virus all over the world.
- The prevalence of PCR positives among close contact screening and age and sex distribution may reflect healthcare access or behavior and this should be analyzed in future studies.
